# A Perturbed MicroRNA Expression Pattern Characterizes Embryonic Neural Stem Cells Derived from a Severe Mouse Model of Spinal Muscular Atrophy (SMA)

**DOI:** 10.3390/ijms160818312

**Published:** 2015-08-06

**Authors:** Andrea Luchetti, Silvia Anna Ciafrè, Michela Murdocca, Arianna Malgieri, Andrea Masotti, Massimo Sanchez, Maria Giulia Farace, Giuseppe Novelli, Federica Sangiuolo

**Affiliations:** 1 Department of Biomedicine and Prevention, University of Rome Tor Vergata, Via Montpellier 1, 00133 Rome, Italy; E-Mails: andrealukk@gmail.com (A.L.); miky.murdi@hotmail.it (M.M.); arianna.malgieri@gmail.com (A.M.); mariagiulia.farace@uniroma2.it (M.G.F.); novelli@med.uniroma2.it (G.N.); sangiuolo@med.uniroma2.it (F.S.); 2 Gene Expression-Microarrays Laboratory, Bambino Gesù Children’s Hospital-IRCCS Polo di Ricerca-V.le di San Paolo 15, 00146 Rome, Italy; E-Mail: andrea.masotti@opbg.net; 3 Department of Cell Biology and Neurosciences, Istituto Superiore di Sanità, 00161 Rome, Italy; E-Mail: massimo.sanchez@iss.it

**Keywords:** survival motor neuron (SMN), spinal muscular atrophy (SMA), neural stem cells (NSCs), motor neurons (MNs), microRNAs (miRNAs)

## Abstract

Spinal muscular atrophy (SMA) is an inherited neuromuscular disorder and the leading genetic cause of death in infants. Despite the disease-causing gene, survival motor neuron (*SMN1*), encodes a ubiquitous protein, *SMN1* deficiency preferentially affects spinal motor neurons (MNs), leaving the basis of this selective cell damage still unexplained. As neural stem cells (NSCs) are multipotent self-renewing cells that can differentiate into neurons, they represent an *in vitro* model for elucidating the pathogenetic mechanism of neurodegenerative diseases such as SMA. Here we characterize for the first time neural stem cells (NSCs) derived from embryonic spinal cords of a severe *SMNΔ7* SMA mouse model. *SMNΔ7* NSCs behave as their wild type (WT) counterparts, when we consider neurosphere formation ability and the expression levels of specific regional and self-renewal markers. However, they show a perturbed cell cycle phase distribution and an increased proliferation rate compared to wild type cells. Moreover, *SMNΔ7* NSCs are characterized by the differential expression of a limited number of miRNAs, among which miR-335-5p and miR-100-5p, reduced in *SMNΔ7* NSCs compared to WT cells. We suggest that such miRNAs may be related to the proliferation differences characterizing *SMNΔ7* NSCs, and may be potentially involved in the molecular mechanisms of SMA.

## 1. Introduction

Spinal muscular atrophy (SMA) is one of the most common autosomal recessive disorders (incidence of 1–5000 to 1–10,000), leading to the progressive degeneration of the α-motor neurons in the spinal cord [1]. The disease is caused by the deletion or mutations of survival motor neuron-1 gene (*SMN1*), resulting in very low levels of functional SMN protein [2]. In humans, *SMN1* localizes on chromosome 5 at an unstable region of 500 kb, which is frequently subjected to rearrangements. Additionally, this region contains a nearly identical copy of the *SMN1* gene, named *SMN2*. *SMN2* harbors a critical difference distinguishing it from *SMN1*, consisting of a single nucleotide substitution in exon 7, translationally silent, which strongly affects the production of the full-length transcript [3,4]. In *SMN2* this results in the transcription of two isoforms, the full length mRNA, encoding the functional SMN protein, and the predominant transcript lacking exon 7 (*SMNΔ7*). The SMNΔ7 protein does not oligomerize efficiently and is rapidly degraded, thus causing a reduction in SMN levels, which are insufficient for the correct function and for the survival of motor neurons [5]. Importantly, SMA disease severity directly correlates with the amount of functional SMN protein detected cellularly [6,7]. SMN is the core of a large macromolecular complex, mediating the biogenesis of small nuclear ribonucleoproteins (snRNPs), the building blocks of spliceosome. In the cytoplasm, the SMN complex chaperones the efficient assembly of Sm proteins around a conserved sequence (Sm site) of small nuclear RNAs (snRNAs), such as U1, U2, U4, U5, U11, U12 and U4atac, generating snRNPs [8]. Although SMN is ubiquitously expressed in all tissues and is essential for cell viability in diverse eukaryotic organisms [9,10], the question of how reduction in SMN levels results in an apparently cell selective motor neuron phenotype remains unexplained [11].

In fact, the hallmark of SMA disease is the selective death of motor neurons, the cholinergic cells of the spinal ventral horns responsible for mediating the central nervous system’s control of voluntary muscle movement. As there are no effective treatments for this devastating disorder, studying the unique biology of these important cells has been the focus of intense research and, at the organismal level, animal models of SMA are of invaluable importance to address the above points. For this reason, several different mouse models have been generated with varying degrees of phenotypic severity [12]. In mice, the *Smn* gene (the equivalent of *SMN1* in humans) is present in single copy and the homozygous loss of *Smn* is embryonically lethal. The presence of two copies of *SMN2* on an *Smn* null background results in mice with SMA that survive for five days after birth [13]. The addition of the human *SMNΔ7* transgene into these mice extends survival to around 14 days, creating the Δ7 SMA mouse model: *Smn^−/−^*, *SMN2^+/+^*, *SMNΔ7^++^* [14], characterized by an early impairment of motor behavior correlated with motor neuron loss. 

One very promising cell model showing great potential for elucidating the neurodevelopmental processes *in vitro* and *in vivo* is that of neural stem cells (NSCs), self-renewing multipotent cells that can be generated from different areas of the developing central nervous system (CNS) [15] and that can be propagated in culture as multicellular structures, called neurospheres, able to differentiate into neurons, astrocytes and oligodendrocytes [16].

Over the last decade, microRNAs (miRNAs), short single-stranded RNA molecules (21–25 nt), were discovered and demonstrated to work as crucial regulators of gene expression at the post-transcriptional level by targeting the complementary sequences in 3′ untranslated regions (3′UTR) of specific mRNAs [17]. MicroRNA regulation is importantly involved in some neurodegenerative states [18,19], and recent studies have shown their function in the control of the timing of many developmental programs, strongly suggesting that these molecules also play an important role in the timely generation of neurons from neural progenitor cells [20]. Recently, few studies have started addressing the role of miRNAs in NSCs, specifically in the developing spinal cord [21,22,23,24] where the lower (α) motor neurons affected by SMA reside. These studies have demonstrated the overall importance of miRNAs in spinal cord development or in motor neuron differentiation and function. However, a more specific experimental approach is required to unravel the role of miRNAs in the neural stem cells resident in the developing spinal cord and committed to yield those motor neurons which are affected in SMA.

To date, *SMNΔ7* NSCs have never been described and characterized, nor have correlations between their genotype, SMN levels and their expression profiles been derived. In this work, we provide a molecular and biochemical characterization of NSCs derived from embryonic (E13.5) spinal cords of *SMNΔ7* mice, comparing them to those derived from their wild type littermates. In particular, we profile the microRNA expression differences distinguishing SMA from WT NSCs, and speculate a possible correlation between some of these microRNAs and the difference in proliferation shown by SMA spinal cord-derived NSCs compared to the wild type.

## 2. Results

### 2.1. SMNΔ7 Stem Cells Derived from E13.5 Spinal Cords form Neurospheres and Express Specific Regional Markers

**Figure 1:**
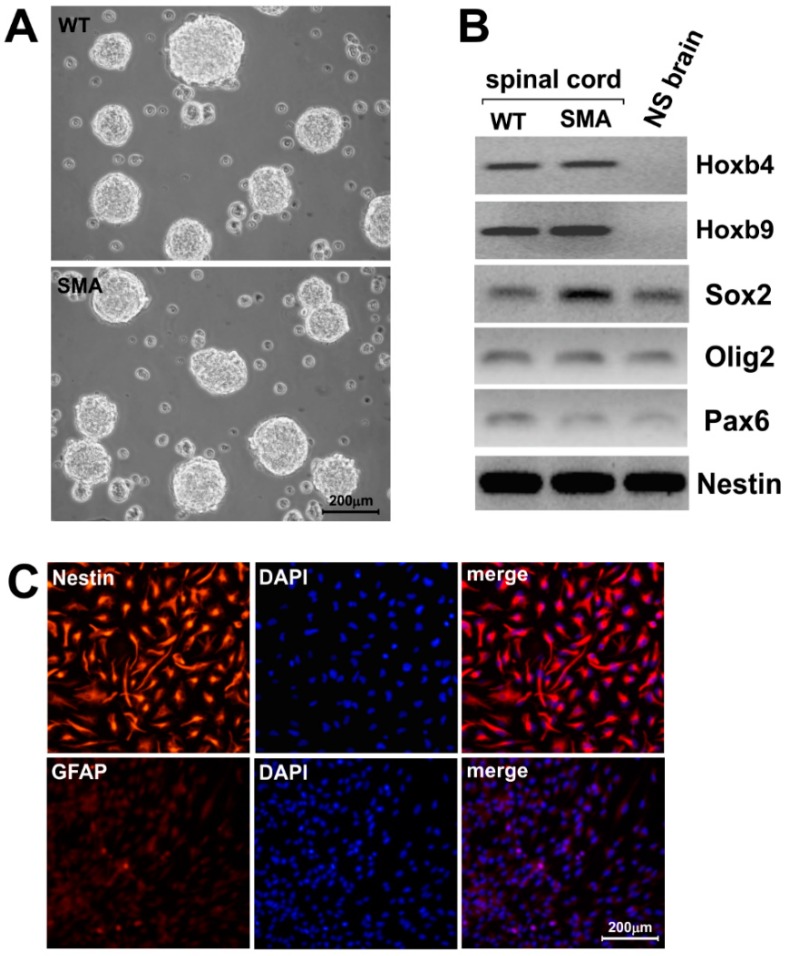
Characterization of NSCs (neural stem cells) derived from *SMNΔ7* SMA (spinal muscular atrophy) and WT (wild type) mice. Representative images of neurospheres isolated from spinal cord of E13.5 SMA and wild type mice, after 2 days of growth. Scale bar: 200 μm (**A**); RT-PCR analysis of two relevant rostrocaudal markers, *Hoxb4* and *Hoxb9*, and a set of characteristic general markers of neural progenitors/radial glia (*Sox2*, *Olig2*, *Pax6* and *Nestin*). The analysis has been performed on ten different animals per genotype and a representative sample for each is shown. The last lane on the right (NS brain) shows the results of the amplification of the same markers, performed on a mRNA sample extracted from neural stem cells derived from the brain of a WT littermate. *Nestin* was used as a loading control for all the RT-PCR amplifications (**B**); Representative images of immunocytochemical analyses of *Nestin*, a neural stem cell-associated marker on wild type NSCs. No signal for the astrocytic marker GFAP (glial fibrillary acidic protein) is present. Scale bar: 200 µm (**C**).

With the purpose of characterizing spinal cord-derived NSCs, we isolated these neural precursors from E13.5 *SMNΔ7* SMA and WT mice, and thus obtained long-lasting cultures of cells growing as neurospheres. Representative images of WT and SMA NSCs are shown in Figure 1A.

As reported in the literature, a characteristic pattern of transcription factors is expressed in each progenitor domain of the central nervous system, belonging to distinct CNS regions [25]. Based on this, RT-PCR analysis was performed, revealing that NSCs derived from spinal cord expressed the anterior transcription factors *Hoxb4* and *Hoxb9* [26] (Figure 1B), while neurospheres derived from the brain of a WT littermate did not. On the contrary, the typical neural progenitor markers *Nestin*, *Sox2*, *Olig2* and *Pax6*, were expressed in all NSC samples, irrespective of the genotypes (WT or SMA) and of the derivation (spinal cord or brain) (Figure 1B).

Self-renewing NSCs homogeneously showed immunoreactivity for *Nestin* and were negative for GFAP (Glial Fibrillary Acidic Protein), a specific marker for astrocytes (Figure 1C). These results confirm that we have successfully isolated specific NSC populations from embryonic spinal cord of *SMNΔ7* SMA mice, maintaining the expression of the respective molecular markers of their original neural environment.

In order to better characterize the *SMNΔ7* SMA mice NSCs, the transcript (Figure S1A) and protein levels (Figure S1B) of murine *Smn* were evaluated in spinal cord-derived NSCs, comparing different genotypes. Protein levels were evaluated in two WT samples named 5 and A, and in three SMA samples (SMA B, SMA 2 and SMA 6). Data obtained confirmed the phenotype-genotype correlation, indicating a strong reduction of murine *Smn* transcript in affected mice respect to wild type ones. A corresponding decrease in protein expression was shown, as also displayed by densitometric analysis (Figure S1B). Additionally, to further characterize *SMNΔ7* NSCs, we employed RT-qPCR to measure the steady state levels of several U snRNAs reported to be affected in SMA mice [27,28,29], and confirmed the alteration of snRNP profile in spinal cord-derived *SMNΔ7* SMA NSCs *vs.* WT NSCs (*p* < 0.05) (Figure S1C). Thus, from this point of view, we have devised a cell model representing an invaluable tool to study the mechanisms of this disorder.

### 2.2. SMNΔ7 Neural Stem Cells Show a Perturbed Cell Cycle Phase Distribution and Increased Proliferation Rate Compared to Wild Type Cells

**Figure 2:**
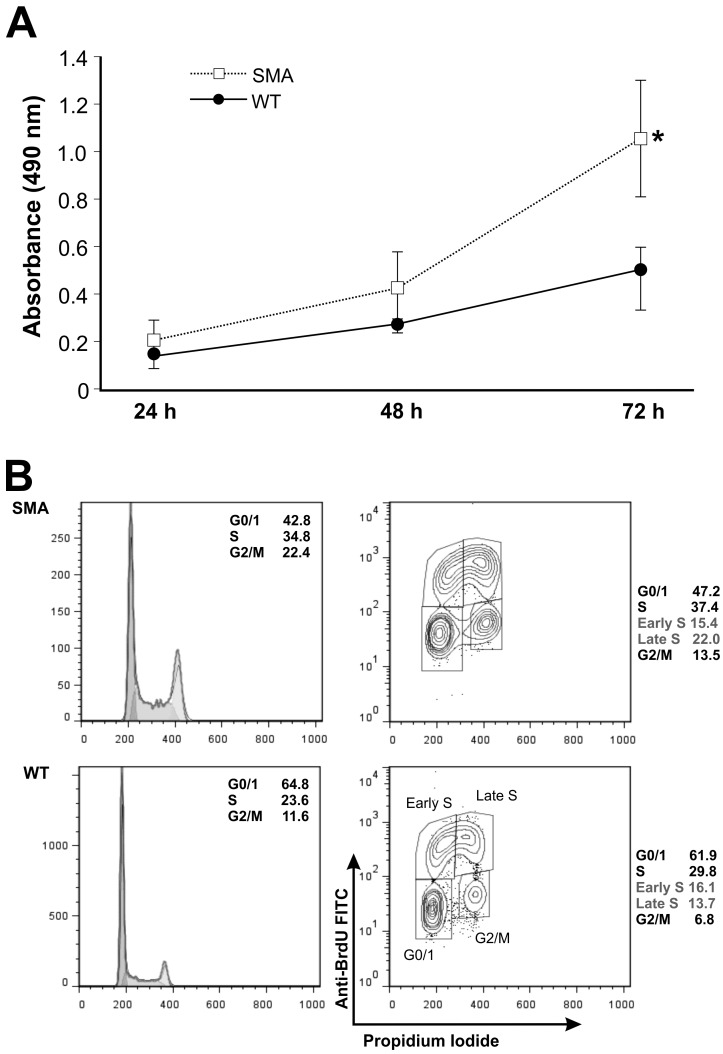
Viability and cell cycle analyses of NSCs from spinal cord. The graph shows the results of a viability (MTS) assay performed on NSCs derived from three animals per genotype, deriving from three distinct families. Each family contributed one wild type and one SMA mouse. Results are shown as the average ± the standard deviation and represent the average of two experiments performed independently. * *p* = 0.022 (**A**); Flow cytometry analysis of BrdU-positive cells of WT and SMA cells incubated with BrdU for 1 h. Representative histograms of cell cycle profiling only are shown on the left, reporting an insert with the relative percentage of cells in G0/G1, S and G2/M phases. On the right, histograms show the BrdU-positive cells, and an arbitrary gating strategy is used to identify cell fractions in early and late S phases. An insert in each panel reports the percentages of cells in the different phases of the cell cycle. Values in (**B**) are representative of one of three independent experiments, whereas the mean values and respective standard deviations are reported in Results section.

With the aim of detecting possible differences in the proliferative capacity of SMA NSCs, we performed viability assays in which we compared the viability of spinal cord-derived SMA neural precursors to WT cells derived from three distinct families. Each family contributed one WT and one SMA mouse. After plating 1 × 10^5^ cells in a 96-well plate, we measured cell viability at 24, 48, and 72 h. In all cases, SMA cells showed a statistically significant (*p* = 0.022) increase in viability at 72 h, likely indicating an enhanced proliferation rate of these cells (Figure 2A). To further investigate this aspect, we performed a cell cycle analysis by flow cytometry. As shown in Figure 2B (histograms on the left), SMA NSCs showed clearly different cell cycle profiles as compared to the WT cell lines. In particular, SMA NSCs showed a higher percentage of cells in S and G2/M phases (34.8% *vs.* 23.6% and 22.4% *vs.* 11.6%, respectively), and less cells in G0/G1 phase (42.8% *vs.* 64.8%), confirming a greater proliferation proficiency, as previously observed in the viability assays (The results shown in histograms on the left of Figure 2 are representative of one of three independent experiments).

Flow cytometry analyses of BrdU labeled cells were performed for a finer characterization of the progression along the cell cycle of NSCs. The distribution in each cell cycle phase of cells labeled with BrdU for 1 h were quantified by using specific gates and the representative data of one of three independent experiments are shown in Figure 2B (right panel). In details, a comparison between SMA NSCs and WT cells highlights a reduced number of cells in G0/1 phase (47.1% ± 0.14% *vs.* 64.5% ± 2.59%) and an increased S and G2/M phases (36.6% ± 0.6% *vs.* 29.2% ± 0.4% and 15.4% ± 1.9% *vs.* 6.6% ± 0.2%), respectively. In addition, S phase was further divided into subregions to distinguish between early and late S phase cells, showing a higher percentage of SMA NSCs in the S phase compared to the WT cells (22.1% ± 0.1% *vs.* 13.2% ± 0.5%), while a quite similar percent of cells was observed in early S phase (14.5% ± 0.9% *vs.* 16.0% ± 0.1%).

Together with the results of the viability assay, these results suggest that SMA NSCs have an enhanced proliferation rate compared to WT NSCs.

### 2.3. The Expression of Specific microRNAs Is Affected in SMNΔ7 Neural Stem Cells

**Figure 3:**
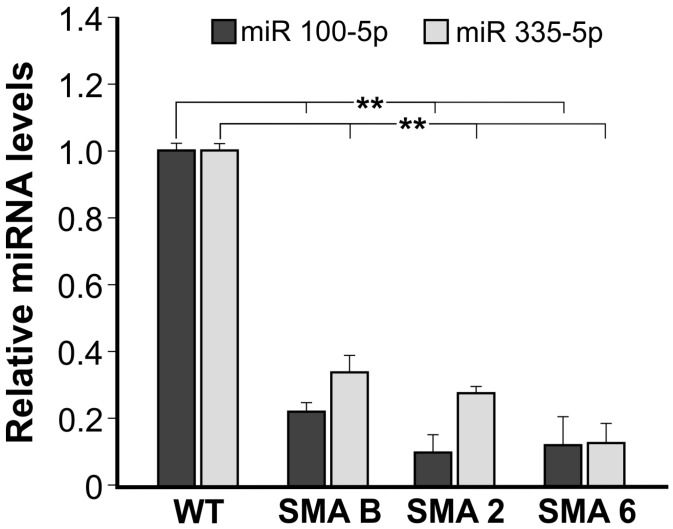
Real-time RT-PCR analyses of miR-100-5p and miR-335-5p. Real-time qPCR of miR-100-5p and miR-335-5p in three distinct NSCs SMA samples (SMA B, SMA 2, SMA 6) derived from spinal cord, using WT sample as references. The data were normalized to internal control small nucleolar RNA234 (snoRNA234). Data are representative of three independent biological replicates; values represent mean ± SD; ** *p* = 0.01.

MicroRNAs are potent modulators of gene expression, and even slight variations in their amounts can strongly affect the production of specific proteins and, consequently, cell fate. In order to determine if microRNAs are involved in the SMA state in neural progenitors, we performed a microRNA expression profiling in three SMA NSC samples, extracted from the spinal cords of mice born in three distinct families. The total RNAs purified from such samples was pooled and compared to the pooled RNAs purified from three WT NSC samples. Among modulated miRNAs, we could confirm the previously described [30] trend of reduction of miR-9-5p (Log_2_ FC_SMA/WT_ = −0.86, *p* = 0.05; data not shown) in SMA samples. We then restricted our analysis to statistically significant (*p* < 0.05) and differentially expressed microRNAs (Log_2_ FC_SMA/WT_ greater than 0.6 for up-regulated and smaller than −0.5 for the down-regulated ones). The list of microRNAs distinguishing SMA from WT NSCs is reported in Table 1. Specifically, 13 miRNAs were underexpressed in SMA NSCs compared to the WT, and only three were overexpressed. Among modulated miRNAs, we focused on miR-335-5p and miR-100-5p, which were highly reduced in SMA NSCs with a Log_2_ FC_SMA/WT_ among the lowest (−1.69 and −1.74, respectively). We validated this result by RT-qPCR not only in the same pooled samples previously submitted to the microarray analysis (data not shown), but also in three distinct SMA NSC samples, named SMA B, SMA 2 and SMA 6, and compared to WT (Figure 3; *p* = 0.01). Results showed that the expression of both miR-100-5p and miR-335-5p in all three SMA NSCs was statistically reduced compared to WT NSCs, further confirming our microarray data.

**Table 1:** MicroRNAs differentially expressed in SMA *vs.* WT NSCs.

**Underexpressed in SMA *vs.* WT**		
**miRNA**	***p*-Value**	**Log_2_ (SMA/WT)**
mmu-miR-20b-5p	4.73 × 10^−3^	−1.81
mmu-miR-6944-5p	2.79 × 10^−2^	−1.78
mmu-miR-100-5p	3.22 × 10^−2^	−1.74
mmu-miR-335-5p	1.70 × 10^−2^	−1.69
mmu-let-7k	4.76 × 10^−2^	−1.31
mmu-miR-6931-5p	3.88 × 10^−2^	−1.15
mmu-miR-1224-5p	3.77 × 10^−2^	−1.13
mmu-miR-3960	1.00 × 10^−2^	−0.81
mmu-miR-26b-5p	1.65 × 10^−2^	−0.76
mmu-miR-2861	3.47 × 10^−2^	−0.72
mmu-miR-7047-5p	2.01 × 10^−2^	−0.52
mmu-miR-106a-5p	4.90 × 10^−2^	−0.52
mmu-miR-23b-3p	3.35 × 10^−2^	−0.51
**Overexpressed in SMA *vs.* WT**		
**miRNA**	***p*-Value**	**Log_2_ (SMA/WT)**
mmu-miR-466q	3.98 × 10^−2^	0.80
mmu-miR-1187	1.32 × 10^−2^	0.64
mmu-miR-466g	2.58 × 10^−2^	0.64

The upper panel lists the microRNAs whose expression is reduced in SMA (spinal muscular atrophy) NSCs (neural stem cells) compared to WT (wild type) ones, while the lower panel shows the microRNAs that are overexpressed in SMA *vs.* WT NSCs. Only statistically significant (*p*-value < 0.05) microRNAs with a Log_2_ FC_SMA/WT_ difference greater than 0.6 or smaller than −0.5 are shown.

## 3. Discussion

The neurosphere system has been used extensively since it is believed to provide a three-dimensional environment that can mimic the neurogenic niche [31]. For this reason, it is considered a valuable *in vitro* model to study neurodevelopmental processes. Moreover, it has a great passage potential compared to adherent monocultures, enabling large scale *in vitro* expansion of neural stem and progenitor cells for drug screening and cell replacement therapy [32].

In this work, we describe for the first time the isolation and characterization of neural stem cells from the spinal cord of a severe *SMNΔ7* SMA mouse model, making comparisons to those isolated from wild type ones.

We also provide an additional aspect to the molecular characterization of our *in vitro* models of progenitor cells involved in SMA, by revealing that a number of miRNAs are modulated in E13.5 *SMNΔ7* NSCs, compared to their respective WT counterparts. 

Among miRNAs modulated in spinal cord SMA NSCs, miR-100-5p is one of the most reduced ones compared to WT cells; the finding that one of the demonstrated targets of miR-100 is insulin-like growth factor 1 receptor (IGF1R) [33] may be relevant in the NSC context, as IGFR1 was shown to promote proliferation of neural progenitors [34]. Thus, the downregulation of miR-100 in SMA NSCs of the spinal cord might be translated into an abnormally high expression of IGF1R, in turn driving excessive proliferation that we observed in our spinal cord NSC model. Indeed, a similar observation was published, reporting an increased rate of proliferation for neurospheres derived from a severe SMA mouse model (*Smn*^−/−^, *SMN2*), and suggesting an inability to appropriately exit the cell cycle [15]. Another miRNA, among those modulated in SMA NSCs compared to WT ones, that mostly drew our attention is miR-335-5p, because of the novelty of this observation, together with some data present in the literature possibly linking this miRNA and the SMA condition. Recently, Dohi and collaborators demonstrated that miR-335-5p is harbored within an intron of its protein-coding host gene, *MEST*, known to be epigenetically regulated through methylation [35], and Maeda *et al*. [36] compared the transcriptome of SMA motor neurons derived from murine embryonic stem cells (mESCs) to that of WT, showing that *MEST* transcript was reduced (1.8-fold) in SMA MNs. Our results about the reduced expression of miR-335-5p in SMA NSCs suggest that also in this context miR-335-5p expression may be directly linked to that of its host gene, and likely subjected to epigenetic regulation. In agreement with this, Schoeftner *et al*. [37] showed that miR-335-5p and *MEST* expression are consistently upregulated during mESC differentiation, where miR-335-5p plays an important role in the regulation of Oct4 and Rb1. The reduced miR-335-5p expression in SMA NSCs derived from spinal cords of E13.5 embryos, support the hypothesis that the SMA condition retains a signature of self-renewal and/or a delay in the upregulation of genes involved in the complex molecular changes eventually leading to cell differentiation.

In conclusion, we have provided a molecular characterization of neural progenitors derived from mouse spinal cord at E13.5, when a peak of neurogenesis takes place, by making comparisons of SMNΔ7 cells, representing a mouse model of severe SMA. We specifically focus on miRNAs differentially expressed in SMA *vs*. WT NSCs, and highlight for the first time the underexpression of miR-100-5p and miR-335-5p in SMA cells compared to those which are WT. The differential expression of these miRNAs may be correlated to the increased proliferation activity observed in *SMNΔ7* cells.

## 4. Experimental Section

### 4.1. Animal Models

Breeder pairs for *SMNΔ7* SMA mice on a FVB background (Stock number 005025; FVB.Cg-Tg (SMN2*Δ7) 4299Ahmb Tg (*SMN2*) 89Ahmb Smn1tm1Msd/J; RRID:IMSR_JAX:005025) were purchased from Jackson Laboratories. Tail-purified DNAs were used for mouse genotyping as described in Le *et al*. [14]. All *in vivo* studies were carried out in accordance with European Economic Community Council Directive 86/109, OJL 358, 1 December 1987 and with NIH Guide for the Care and Use of Laboratory Animals.

### 4.2. Isolation and Culture of Neural Stem Cells (NSCs)

NSCs were isolated from brains and spinal cords of *SMNΔ7* SMA and WT mice at E13.5. Isolated tissues were mechanically dissociated with a pipette and incubated in 0.05% trypsin/EDTA solution for 15 min at 37 °C. Single-cell suspension was seeded at a density of 100,000 cells/mL in Neurobasal medium (Gibco, Life Technologies Corporation, Carlsbad, CA, USA) supplemented with 2  mM l-Gln (Euroclone, Milan, Italy), 100 U/mL penicillin-streptomycin (Euroclone), 1× N_2_ supplement (Gibco), 1× B27 Supplement (Gibco), 20  ng/mL murine EGF and FGFbasic (Prepotec, London, UK). Cells were grown in uncoated T25 plastic flasks as free floating clusters and passaged every 5–7 days.

### 4.3. RT-PCR Analysis

Total RNA was extracted from NSCs with Trizol Reagent (Invitrogen, Life Technologies Corporation, Carlsbad, CA, USA) according to the manufacturer’s recommendations. One microgram of total RNA, DNAse I treated for eliminating genomic DNA contamination (Ambion, Life Technologies Corporation, Foster City, CA, USA), was reverse transcribed using High-Capacity cDNA Archive kit (Life Technologies Corporation, Foster City, CA, USA) and amplified using primers reported in Table 2. Specific amplification conditions for each marker were as previously reported [25]. *Nestin* was used as loading control for testing the efficacy of all the amplification reactions.

**Table 2:** Oligonucleotide sequences.

Gene	Forward	Reverse
*Gapdh*	CATGGCCTTCCGTGTTCCTA	GCGGCACGTCAGATCCA
*Hoxb4*	TCACGTGAGCACGGTAAACCC	GCGTCAGGTAGCGATTGTAGTGAA
*Hoxb9*	ATTTGCGAAGGAAGCGAGGACA	TAGCTCCAGCGTCTGGTATTTGGT
*Olig2*	GTTCTCCTCCGCAGCGAG	CCTTCTTTTTTCAACCTTCCGA
*Pax6*	CAGCTCCAGCATGCAGAACA	CCGCCCGTTGACAAAGAC
*Sox2*	AGAAGAGGAGAGAGAAAGAAAGGGAGAGA	GAGAGAGGCAAACTGGAATCAGGATCAAA
*Nestin*	AGGCTGAGAACTCTCGCTTGC	GGTGCTGGTCCTCTGGTATCC
*U1*	CCTGGCAGGGGAGATACCATGAT	TGCAGTCGAGTTTCCCGCATTT
*U2*	CGGCCTTTTGGCTAAGATCAAGTG	TCCTCGGATAGAGGACGTATCAGA
*U4*	GAGGTTTATCCGAGGCGCGATTAT	CACGGCGTATTGGGAAAAGTT
*U11*	CGTGCGGAATCGACATCAAGAGA	CAACGATCACCAGCTGCCCAATTA
*U12*	AACTTATGAGTAAGGAAAATAACGATTCG	CCGCYCAAAAATTCTTCTCACA
*U4atac*	TTTCTTGGGGTTGCGCTACTGT	AAAGCAGAGCTCTAACCGATGCAG
*5.8S*	GCGCTAGCTGCGAGAATTAAT	CAAGTGCGTTCGAAGTGTCGA
*SMN FL*	TGCTCCGTGGACCTCATTTCT	TGGCTTTCCTGGTCCTAATCC

### 4.4. Expression Analyses by RT-qPCR

Total RNA from SMA and WT NSCs was extracted with Trizol Reagent (Invitrogen, Life Technologies Corporation) following manufacturer’s instructions. Genomic DNA contamination was eliminated with DNase I-RNase-free (Ambion, Life Technologies Corporation). One microgram of RNA was reverse transcribed with the High-Capacity cDNA Archive kit (Life Technologies Corporation) and analyzed by RT-qPCR, following an amplification protocol specific for each primer pairs. mRNAs of interest were quantified with specific primers (Table 2) using SYBR Green chemistry (Life Technologies Corporation). The comparative ΔΔ*C*_t_ method was used to quantify relative gene expression levels [29,38] and normalization was performed by simultaneous quantification of *Gapdh* (Glyceraldehyde 3-phosphate dehydrogenase) mRNA.

### 4.5. MTS Cell Viability Assay

The CellTiter 96 AQueous One Solution cell proliferation assay kit (Promega, Madison, WI, USA) was used to assess the number of viable cells following the manufacturer’s instructions. The neurospheres were manually dissociated and 1 × 10^5^ cells were plated in each well of a 96-well plate, in triplicate. After 24, 48 or 72 h, twenty microliters of MTS reagent were added to cells in 100 microliters medium per well in 96-well plates. After incubation at 37 °C (5% CO_2_) for 2 h, viable cells were measured at 490 nm absorbance in an ELISA Bio-Rad 680 spectrophotometer (Bio-Rad, Hercules, CA, USA). Background absorbance was recorded with the medium (DMEM).

### 4.6. Flow Cytometry

Murine derived NSCs (WT, SMA) were disgregated as single cells for flow cytometry analysis. Cells were grown free floating for 24 h in culture medium described above at 5% CO_2_ and 37 °C. After 24 h cells were incubated for one hour with 10 microM BrdU (Life Technologies Corporation) added in the culture medium. After treatment cells were collected and centrifuged for 5 min at 700 rpm. Cells were washed twice with washing buffer (PBS + 1% *w*/*v* BSA). Pelleted cells were resuspended with 300 microliters of PBS, put on ice and fixed by adding 700 microliters of cold ethanol 90%. Cells were incubated for 30 min on ice and then collected by centrifugation for 10 min at 2000 rpm. Ethanol was removed and pellet was resuspended by gentle vortexing, followed by addition of 1 mL of 2 N HCl + 0.5% Triton X-100. Cells were incubated for 30 min at room temperature and then centrifuged for 3 min at 2200 rpm. Pelleted cells were resuspended with 1 mL of 0.1 M borate buffer and centrifuged for 5 min at 2200 rpm. Two washes with 1 mL of washing buffer were performed and cells centrifuged for 5 min at 2200 rpm. Cells were resuspended with 200 microliters of washing buffer + 1:50 mouse anti-BrdU antibody (Invitrogen, Life Technologies Corporation, CAT#33900, RRID:AB_86146) and incubated for 45 min at room temperature followed by two washes. Subsequently cells were incubated with FITC-conjugated secondary antibody (1:800) for 30 min at +4 °C. For DNA content assay, cells were successively stained with propidium iodide (Sigma-Aldrich, St. Louis, MO, USA). Cytofluorimetric acquisition was done on a BD FACScalibur using CellQuest software (BD Biosciences, San Jose, CA, USA), analyses were performed using FlowJo software v. 7.6.5 (Tree Star, Inc., Ashland, OR, USA).

### 4.7. miRNA Microarray Assay

Total RNA isolated from three distinct NSC cultures derived from three E13.5 SMA embryos and from three WT embryos was extracted using TRIzol (Invitrogen), according to the provided protocol. The quantity and quality of the isolated RNA were assessed by the Agilent 2100 Bioanalyzer™ (Agilent Technologies, Foster City, CA, USA) and by spectrophotometer using 260/280 nm and 260/230 nm ratios. The RNAs deriving from embryos of the same genotypes were pooled and 5 micrograms of each pool was subjected to microarray analysis. The miRNA microarray assay was performed by LC Sciences (http://www.lcsciences.com; Houston, TX, USA) on µParaflo™ microfluidic chips. 1892 unique probes for mature mouse miRNAs were assayed, based on Sanger miRBase Release 20. Background was subtracted from raw data, and the signal value for each probe was considered to be detectable if it met 3 conditions: (1) signal intensity more than 3 times the background standard deviation; (2) spot coefficient of variation (*CV*) < 0.5, where *CV* = standard deviation/signal intensity; and (3) microRNAs listed as detectable if at least 50% of its repeating probes were above the detection level. Detectable signals were normalized to remove system-related variations using a cyclic loess approach [39]. Statistical comparison between various groups was performed by Student’s *t-*test for independent samples, as appropriate. Differences were considered significant when *p* values were less than 0.05.

### 4.8. Validation of miRNA Expression

Total RNA, extracted as reported above, was reverse transcribed using the miR-335 and miR-100 TaqMan MicroRNA Assays (Life Technologies Corporation). The small nucleolar RNA (snoRNA234) was used as reference. The quantitative stem-loop real time polymerase chain reaction (qPCR) was performed according to conditions suggested by Life Technologies.

### 4.9. snRNP (Small Nuclear Ribonucleoproteins) Level Analysis

snRNAs were quantified by SYBR Green chemistry Real Time qPCR (Power SYBR Green PCR Master Mix, Life Technologies Corporation). Total RNA from NSC SMA and WT was purified as described above. 5.8S rRNA, used as a reference, and snRNA specific reverse primers (see Table 2) were used to generate cDNA, using High-Capacity cDNA Archive kit (Life Technologies Corporation). The same reverse primers were used in both RT-PCR and qPCR.

### 4.10. Immunofluorescence

A set of neurospheres were trypsinized to obtain a suspension of dissociated cells. These cells were then plated in tissue culture plates pre-coated with poly-d-lysine (PDL, Sigma-Aldrich, St. Louis, MO, USA) and fixed after 4 h with 4% paraformaldehyde in PBS. Specific dilution of primary antibodies were used: Nestin 1:200 (Chemicon International, Inc., Billerica, MA, USA, CAT#AB5922, RRID:AB_91107) and GFAP 1:100 (Sigma, CAT#5-A-5, RRID:AB_2314539). Cells were subsequently incubated for 1 h at room temperature with specific secondary antibodies (1:800 Alexa Four 568, 488 conjugated, Molecular Probes, Life Technologies Corporation), and DAPI (1:1000, Invitrogen, Life Technologies Corporation).

### 4.11. Western Blot Analysis

NSCs were homogenized in RIPA buffer (50 mM Tris:HCl pH 7.4; 150 mM NaCl; 1% Triton X-100; 1% sodium deoxycholate; 0.1% SDS), containing complete Mini EDTA-free Protease Inhibitor Cocktail Tablets (Roche, Basel, Switzerland). Total protein extracts were resolved on 12% SDS-PAGE gels and analyzed by Western blot. Nitrocellulose membranes were saturated in 5% milk/PBS and then probed with antibodies against SMN (1:10,000, CAT#610647, BD Transduction Laboratories, RRID:AB_397973, Lexington, KY, USA) and actin (1:400, CAT#sc-1616, Santa Cruz Biotechnology, CA, USA, RRID:AB_10160631), as reference. Peroxidase-conjugated secondary antibodies were used (1:10,000, Millipore, Billerica, MA, USA) and signal was detected with an ECL detection kit (Amersham, GE Health Care, Fairfield, CT, USA).

Densitometric analyses were carried out using the freely available Image J software according to standard procedures.

### 4.12. Statistical Analyses

Statistical significance was determined by ANOVA and *post-hoc* was performed using Bonferroni test or Student’s *t*-test when appropriate. Differences were considered significant when *p* values were less than 0.05 (indicated by * for *p* < 0.05, by ** for *p* < 0.01 and by *** for *p* < 0.001).

## Supplementary Materials

Supplementary materials can be found at http://www.mdpi.com/1422-0067/16/08/18312/s1.

## Abbreviations

SMA: Spinal Muscular Atrophy; SMN: Survival Motor Neuron; MNs, motor neurons; NSCs: Neural Stem Cells; miRNAs: MicroRNAs; snRNPs: Small nuclear ribonucleoproteins; snRNAs: Small nuclear RNAs; 3′UTR: 3′Untranslated regions; WT: Wild Type; IGF1R: Insulin-like Growth Factor 1 Receptor; RT-PCR: Real time polymerase chain reaction; GFAP: Glial Fibrillary Acid Protein; mESC: murine embryonic stem cell.
