# Supplementary material for: A Perturbed MicroRNA Expression Pattern Characterizes Embryonic Neural Stem Cells Derived from a Severe Mouse Model of Spinal Muscular Atrophy (SMA)
Source: Int J Mol Sci. 2015 Aug 6;16(8):18312–27. doi: 10.3390/ijms160818312 (PMC4581247; doi:10.3390/ijms160818312)
Supplement: Supplementary file 1 [file ijms-16-18312-s001.pdf]

Supplementary Information

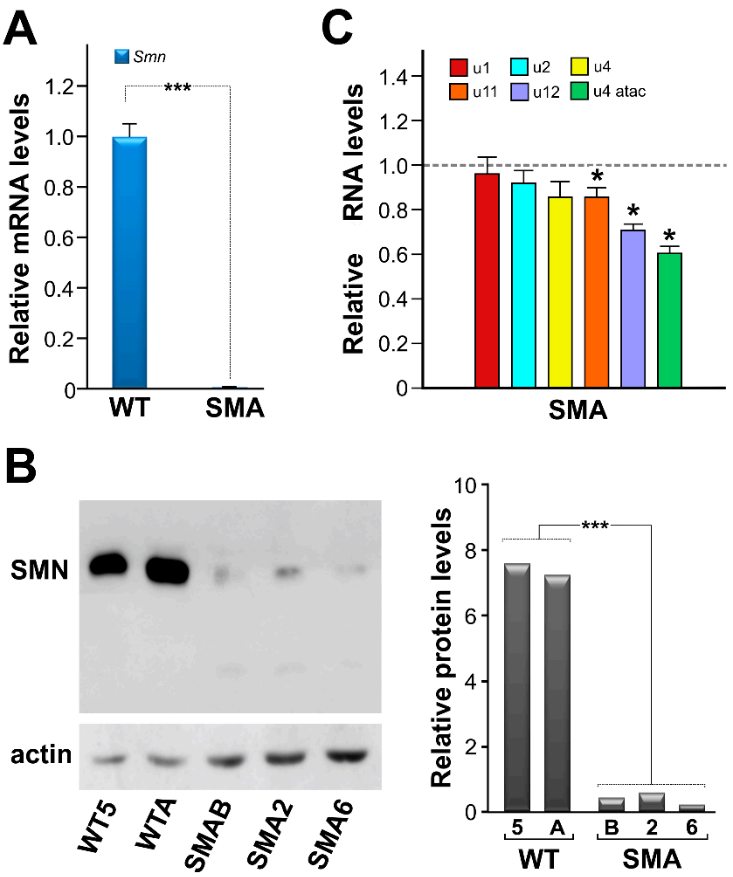

Figure S1. Transcription, protein levels and activity of SMN (survival motor neuron) in spinal cord derived NSCs (neural stem cells). RT-qPCR analyses of murine *Smn* were carried out on WT (wild type) and SMA (spinal muscular atrophy) spinal cord NSCs, setting the expression value of WT NSCs equal to 1; *Gapdh* mRNA was used as a reference gene for data normalization. Data are representative of three independent biological replicates. Values represent mean ± SEM; *** $p < 0.001$. The analysis was performed on NSCs derived from three animals per genotype, deriving from three distinct families (**A**); Western blot analysis of equal amounts of proteins extracted from different NSCs derived from spinal cords of two WT and three different SMA samples. Relative quantification of SMN levels was obtained by densitometric analysis using actin as the loading control; *** $p < 0.001$ (**B**); Total RNAs from WT and SMA spinal cord NSCs were investigated by RT-qPCR for the levels of specific snRNAs. The dashed grey line corresponds to snRNA levels of WT samples, used as reference. Data are representative of three independent biological replicates. The analysis was performed on NSCs derived from three animals per genotype, deriving from three distinct families. Values represent mean ± SEM. * $p < 0.05$ (**C**). SEM: standard error of the mean.
